# On-chip sub-terahertz surface plasmon polariton transmission lines with mode converter in CMOS

**DOI:** 10.1038/srep30063

**Published:** 2016-07-21

**Authors:** Yuan Liang, Hao Yu, Jincai Wen, Anak Agung Alit Apriyana, Nan Li, Yu Luo, Lingling Sun

**Affiliations:** 1 School of Electrical and Electronic Engineering, Nanyang Technological University, 639798 Singapore; 2 Key Laboratory of RF Circuits and Systems, Ministry of Education, and Zhejiang Provincial Laboratory of Integrated Circuit Design, Hangzhou Dianzi University, Hangzhou, 310018, China

## Abstract

An on-chip low-loss and high conversion efficiency plasmonic waveguide converter is demonstrated at sub-THz in CMOS. By introducing a subwavelength periodic corrugated structure onto the transmission line (T-line) implemented by a top-layer metal, surface plasmon polaritons (SPP) are established to propagate signals with strongly localized surface-wave. To match both impedance and momentum of other on-chip components with TEM-wave propagation, a mode converter structure featured by a smooth bridge between the Ground coplanar waveguide (GCPW) with 50 Ω impedance and SPP T-line is proposed. To further reduce area, the converter is ultimately simplified to a gradual increment of groove with smooth gradient. The proposed SPP T-lines with the converter is designed and fabricated in the standard 65 nm CMOS process. Both near-field simulation and measurement results show excellent conversion efficiency from quasi-TEM to SPP modes in a broadband frequency range. The converter achieves wideband impedance matching (<−9 dB) with excellent transmission efficiency (averagely −1.9 dB) from 110 GHz–325 GHz. The demonstrated compact and wideband SPP T-lines with mode converter have shown great potentials to replace existing waveguides as future on-chip THz interconnects. To the best of the author’s knowledge, this is the first time to demonstrate the (sub)-THz surface mode conversion on-chip in CMOS technology.

Meta-devices are recently found as key technology to shape the future realization of high speed, low power on-chip communication as they can be deployed to manipulate EM-field to provide extraordinary performances at THz. Due to the negative permittivity behavior^1^,^2^,^3^,^4^,^5^,^6^,^7^,^8^,^9^, surface plasmon polaritons (SPP), defined as collective oscillations of the delocalized electrons in metal surfaces, forming surface-wave. Owing to their ability to confine light in a subwavelength scale with high intensity, SPPs can be used to miniaturize electronic components at Tera-scale, and build highly integrated circuits. Terahertz wave, which has a wavelength of approximately half a millimeter, can thus be concentrated by a factor of nearly 100 (centimeter at chip and board scale) to travel through metal of copper in standard CMOS process without altering the process and material. Therefore, CMOS integrated circuits with plasmonic technology could operate at much faster speeds than current electronics (nearly the speed of light), while 100 times thinner than glass fibers. This could lead to faster, thinner, lighter and cheaper scalable communication technologies.

For the far-infrared, THz, and microwave frequency bands, metals behave akin to perfectly electrical conductors (PECs), and thus surface plasmon polaritons cannot be supported by a metal surface. To overcome this difficulty, in Pendry *et al*.^2^ suggested for the first time that by using a metamaterial constructed with patterning the metal surface with subwavelength periodic features, one can achieve ‘spoof’ surface plasmons (SSP) with subwavelength confinement at infrared wavelengths and beyond, which mimic surface plasmons at much shorter wavelengths^2^. This metamaterial, or meta-surface approach, offers possibilities of bringing most of the advantages associated with traditional surface plasmon polaritons, to lower frequencies, such as the high confinement of light and compatibility of devices. In particular, in the terahertz and microwave spectrum, where the loss of metal is negligible, devices supporting SSP can achieve higher efficiency and longer propagation length as compared to conventional surface plasmon polaritons. Moreover, in meta-devices, the behaviour of SSP depends primarily on the geometry of the corrugations instead of the optical properties of the metal. This gives us extended freedom in controlling the performance of devices. Later on, Cui *et al*. has demonstrated the propagation of SSP on ultrathin and flexible films to long distances in microwave frequencies^9^. Most recently, we have made further steps to demonstrate the supported SSPs at THz frequency range in standard CMOS process^1^,^10^,^11^,^12^. In integrated circuits, it commonly happens that two interconnects are tightly packed with deep-subwavelength separation but prone to a high EM crosstalk. Our previous work has demonstrated that SSP signals in TM-mode can propagate on chip at mm to cm scale. Moreover, the two SSP transmission lines have much less mutual coupling (19 dB reduction) than the conventional signals in TEM-mode on two coupled microstrip lines with the same size and separation at THz frequencies. Hence it can dramatically suppress the interference in very compact space, providing a potential solution to overcome the challenge of signal integrity for Tera-scale chip level communication. All these theoretical and experimental works have provided important stepping stones for building meta-device on chip. However, all these individual metamaterial require mode conversion block as other on-chip module may process the conventional TEM wave, i.e. the surface-wave needs to be smoothly converted to TEM wave or vice versa to achieve complete compatibility among all on-chip building blocks. For example, for a CMOS THz I/O link (shown in Fig. 1), which governs the surface-wave radiation at transmitter side, requires a high efficiency conversion from TEM wave to surface-wave, because THz source is generated on-chip in the form of TEM wave. Without mode conversion, the transmission of SSP coupler proposed in our previous work has over 3 dB loss at around 300 GHz. However, it is non-trivial to design a mode converter at THz in CMOS. The high-index lossy silicon substrate desensitizes spatial field confinement of SPPs, making conversion difficult. Converter designs reported in refs 13, 14, 15 were verified only in PCB level with bulky size with loss suitable only for GHz applications. For example, a huge grounding geometry is required but is not feasible for a chip level design in CMOS.

In this paper, an on-chip mode converter structure featured by a linearly fading GCPW and gradient groove is reported to achieve both impedance and momentum matching at (sub)-THz frequency. The underlying ground plane is necessary to prevent electrical fields from radiating into the high index substrate. The dual sides ground of GCPW ensures impedance matching when quasi-TEM wave is injected into the structure while the gradient groove serves to mode evolution with smooth transformation of momentum. Distinct from the conventional gradient-index metasurface reported in refs 16, 17, 18, 19, 20, 21, 22, 23, 24, here a novel and compact planarly gradient groove with underlying ground is developed for on-chip mode conversion in CMOS. The converter structure has no need of the GCPW structure and hence is much more compact. As such, the THz signal can be easily fed without complex wave incident approaches refs 16, 17, 18, 19, 20, 21, 22, 23, 24. Near-field and *S* parameters simulation results are given to show the high conversion efficiency achieved by the proposed converter. The converter structure is designed and fabricated in standard 65 nm CMOS technology. Experimental results demonstrate the broadband high efficient transmission of averagely −1.9 dB with good impedance matching (<−9 dB) from 110 GHz to 325 GHz. The transmission is obviously better than conventional on-chip waveguide structure such as microstrip, CPW and GCPW in (sub)-THz band. This work is critical for the future realization of ultra-high speed electronics by THz plasmonic technology.

## Results and Discussion

### SPPs on chip in THz region

The surface plasmon polariton (SPP) propagating at the flat interface between a real metal and a dielectric are naturally 2D electromagnetic waves. Confinement of EM wave is realized since the propagation constant is greater than the wave vector *k* within the dielectric, resulting in evanescent decay on both sides of the interface^25^,^26^,^27^,^28^,^29^,^30^,^31^,^32^. Using localized surface plasmons in flat metals, EM energy could be confined or squeezed into volumes much smaller than the diffraction limit (λ_0_/2n)^3^, where 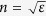 is the refractive index of the surrounding medium. Such a high confinement results in an accompanying field enhancement and is of prime importance in THz transmission. However, care must be taken when qualifying energy confinement, because a subwavelength field decay length on the dielectric side of the interface implies that a substantial amount of the total *E*-field energy of the SPP mode resides inside the metal. From our previous study in ref. 1, the field decay length can be well governed by geometry means, given that no obvious substrate attenuation or reflection occurs in the frequency range of interest. However, in the real design one must seek to resolve these two issues and maintain high efficiency propagation of SPP on metal surface.

**Figure 2:**
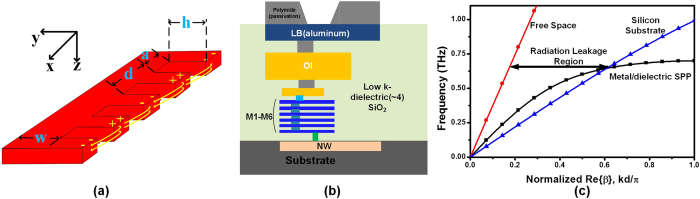
(**a**) The layout and *E*-field distribution of the on-chip SPP T-line, parameters *d*, *h*, *a*, *w* denotes the periodic pitch, groove depth, groove width and line width of SPP T-line, respectively, (**b**) metal configuration of back-end-of-line (BEOL) in standard 65 nm CMOS technology, and (**c**) simulated dispersion diagram considering the high index substrate effect.

**Figure 3:**
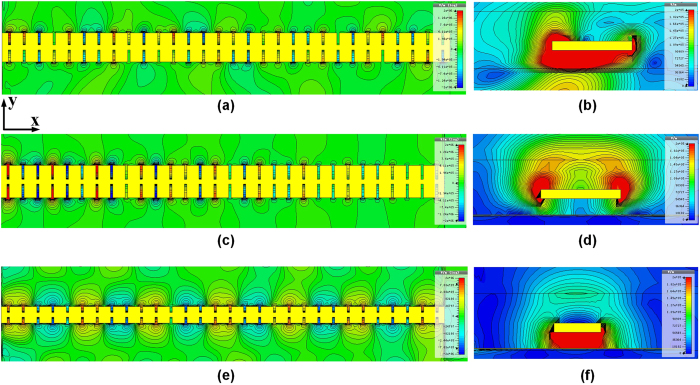
(**a**,**c**,**e**) The simulated amplitude of *E*-field distribution of the designed single SPP T-line (**a**) without underlying ground M1; (**c**): with underlying ground M1, and *h* = 12 μm; (**e**): with underlying ground M1, and *h* = 6 μm) evaluated at the *xy* plane using CMOS process, (**b**,**d**,**f**) *E*-field distribution on the cross-section of the corrugated metal strip evaluated at *yz* plane corresponding to the structure shown in (**a,c,f**), respectively. All simulations are performed at 2 THz.

Apart from Ohmic loss that commonly observed for metals, the propagation length of surface plasmons, which is defined by *L* = (2*im*(*β*))^−1^, where *im*(*β*) is the imaginary part of the propagation vector of the bound modes, has a trade-off between the confinement of subwavelength field for the SPP T-line structure shown in Fig. 2a 1,9, i.e., the stronger of *E*-field localization normally accompanies the shorter propagation length. This is true only if the absorption loss dominates the total loss of surface plasmon excited in the metal/dielectric interfaces. For conventional SPP T-line structures implemented in free-space or low-index substrate board with GHz operation, this situation is not difficult to realize. For example, ref. 9 demonstrate that the propagation efficiency of SPP was as high as 98% after traveling over eight free-space wavelengths. It turns outs that most of EM energy exit as SPP mode and the remaining loss may account for the metal Ohmic loss, while the radiation loss is obviously negligible. However, this is no longer true for surface plasmonic waveguide building onto high index substrate with low resistivity. From circuit’s perspective, the silicon substrate with 70 S/m conductivity forms a low impedance path for SPP mode to radiate into it and attenuates with loss proportional to 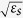, introducing high loss at high frequencies. The typical CMOS metal configuration is illustrated in Fig. 2b, in which the top copper metal is only 4∼5 μm away from the substrate. To evaluate the dispersion property, one unit-cell of planar surface plasmonic waveguide (Fig. 2a, the metal is modeled as PEC) is simulated using CST Microwave Studio by eigenmode calculation in different substrate, in which the wave vector is extracted. Shown in Fig. 2c, the dispersion curve of SPPs excited at the air/metal interface lies outside the light line is defined by *k* = *n*_*air*_***ω***/*c*, and the SPP modes encounter no radiation loss into the air. However, radiation loss takes place at all points of the dispersion curve that lie to the left side of the light line of the substrate *k*_*s*_ = *n*_*s*_***ω***/*c*. As such, for SPP operation frequency lower than *k*_*0*_*n*_*s*_ but larger than *k*_*0*_, radiation loss is inevitable. As on-chip interconnect, SPP T-lines need to operate at frequency somewhat higher than *k*_*0*_ so that it presents slow-wave effect with field localization for low loss transmission, while it should avoid the region higher than *k*_*0*_*n*_*s*_ as the propagation length turns out to be extremely small. As such, in most on-chip communication the operation frequency of SPP T-lines lies into the region that radiation loss by substrate must occur. To evaluate this effect, Fig. 3a illustrates the *E*-field intensity distribution at the *xy* plane for SPP T-line structure shown in Fig. 2a at 2 THz. The periodic pitch *d* is chosen as 12.4 μm and the grooves depth *h* is 12 μm, both of which are much shorter than wavelength (≈150 μm for *f* = 2 THz). The electromagnetic wave is injected directly from the left side of structure while the right side of it terminated by 50 Ω impedance. Clearly, the surface-wave are now tightly confined into the periodical grooves at the beginning with evanescent wave decaying in the *y*-direction but soon fades out along the *x*-direction within several wavelength. However, it turns out that in addition to absorption loss (due to air/metal interface electron dampling), the radiation loss by lossy substrate now dominates the loss mechanism of SPPs propagation. Such a situation is further confirmed by Fig. 3b evaluated at the *xz* plane, in which the localized wave is clearly absorbed by the substrate and the *E*-field are now more likely to crowd next to the substrate. Therefore, on-chip SPPs propagation needs to take radiation leakage into consideration.

While ground plane is not desired in board level design due to integration concern, it becomes quite easy for CMOS integration. For example, the on-chip back-end-of-line (BEOL) configuration in Fig. 2b exhibit 6 copper metal layer each with 0.22 μm thickness, and the bottom metal M1 is feasible as ground plane as it is the farthest away signal trace that is generally realized using the top most copper metal. With M1 as ground plane, one SPP T-line has been demonstrated in ref. 1 for sub-THz crosstalk reduction by surface plasmonic techniques with measured result showing low loss transmission while guiding SPP mode efficiently. However, the groove depth *h* is 6 μm, and therefore the field confinement is not tight at low frequency.

Note that the geometry parameters are key engine determining the guiding property of SPP^33^,^34^,^35^,^36^,^37^,^38^, increasing the groove depth *h* leads to a decrease of the asymptote frequency by observing the dispersion diagram (will be shown later). As such, with deeper grooves the dispersion curves are more bended away from the light line, resulting in smaller wave velocity at low frequencies. Considering the transverse-magnetic (TM) polarized waves confined at the surface of periodical grooves of SPP T-line with parallel momentum *k*_*x*_, with subwavelength condition of λ ≫ *a* and λ ≫ *d*, the momentum along the *x* direction can be approximated by 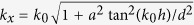
26. For shallow grooves the wave vector simply degenerates to *k*_*0*_ which is the wave vector in free space, and the confinement of SPP vanishes. For deep grooves, however, the momentum of TM-polarized wave are much larger than *k*_*0*_, causing momentum mismatch if a plane TEM wave is guided injecting into the structure. Even though the impedance mismatch has been demonstrated small in sub-THz region by one side groove of SPP T-line in CMOS^1^, momentum mismatch leads to a low transmission efficiency of SPPs. To evaluate such effect, Fig. 3c–f illustrate the *E*-field distribution of two SPP T-line in *xy* plane with *h* = 6 μm and 12 μm, respectively, all simulated at 2 THz. A plane TEM wave with momentum *k*_*0*_ normally used for driving on-chip transmission lines is considered as excitation source. As shown in Fig. 3c, the confinement of *E*-field by the subwavelength grooves demonstrates the fundamental property of SPP modes, and the bound modes are tightly localized with a strong decay along the *y*-direction, which is further confirmed by the *E*-field distribution evaluated at the *xz* plane shown in Fig. 3d. Obviously, the *E*-filed is mainly confined by the grooves, and the ground plane exclusively prevents the field from absorbing by the high index substrate. The color scale from the greatest field strength (red and blue, for positive and negative phase, respectively) to the lowest (yellow) indicates a strong field confinement achieved by an on-chip SPP T-line. However, the bound modes can only propagate up to several wavelength toward *x*-direction, demonstrating low transmission efficiency. As the parameters chosen introduces negligible impedance mismatch at such frequency^1^ (no significant reflection can be observed from Fig. 3c as well), the low transmission efficiency is mainly attributed by momentum mismatches between *k*_*0*_ and *k*_*x*_ at the interface. Note that the modes momentum *k*_*x*_ is geometrically dependent on the grooves, halving the groove depth *h* means a weaker modes confinement by the subwavelength grooves. As shown in Fig. 3e with the same color scale, the *E*-field are now propagating over more than 10 wavelengths along the grooves with much weaker field confinement. The bound mode is not tightly localized by the grooves and hence the polarization almost maintains its original states as it injects into the structure. This could be readily verified by Fig. 3f, in which the *E*-field is evaluated at the *xz* plane. Apparently, compared with Fig. 3d the *E*-field distribution is mainly restricted between the two conductors, akin to that of a microstrip line with underlying ground. As such, for a bare SPP T-line with TEM excitation, there is a trade-off between field confinement and transmission efficiency.

### Design of on-chip SPP T-Line with mode converter at THz

Owing to the constrain of plane metals in CMOS technology, the excitation of TM-polarized wave on-chip is not trivial, unless a surface-wave oscillation could be developed. As such, a smooth conversion between the plane TEM wave (which is normally referred to the guided wave as well) with momentum *k*_*0*_ to the bound mode with momentum *k*_*x*_ becomes mandatory. Conventional converter designs in board level^13^,^14^,^15^ demonstrated the effectiveness of mode converter in microwave region. In their works, converter structures featured by a smooth bridge between the coplanar waveguide (CPW) with 50 Ω impedance and SPP T-line was presented, and both impedance and momentum matching maintain up to 12 GHz due to the smooth transition. A mode converter connecting a conventional microstrip transmission line to the slow-wave Spoof Surface Plasmon (SSP) transmission line was subsequently presented by using a ground regulator to match the momentum at the injection interface^14^. However, these two approaches consume too much of area and the ground plane is outside the region of the SPP T-line, which is not suitable for on-chip implementation at mm-wave to THz frequency. On the other hand, their proposed flaring ground with exponential regulation cannot be fabricated in CMOS technology. To the author’s best knowledge, such a surface plasmonic converter has not been reported so far operating at (sub)-THz in CMOS technology.

**Figure 4:**
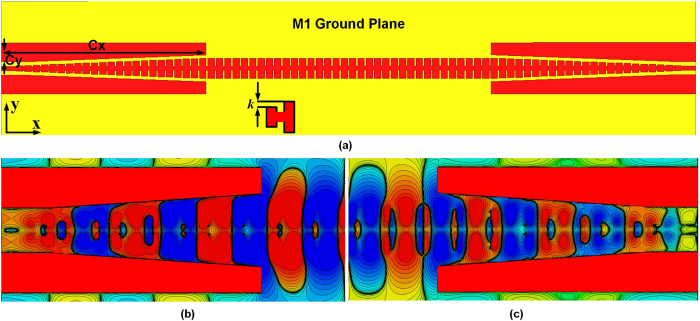
(**a**) The proposed surface plasmonic waveguide with mode converter structure featured by the combination of a linearly fading GCPW with gradient grooves. Parameter *C*_*x*_, *C*_*y*_ and *k* denotes effective GCPW length, effective vertical fading distance, and gradient factor (unit: μm), (**b,c**) the *E*_*x*_ component evaluated at the *xy* plane demonstrating the mode evolution by the proposed converter structure.

To construct an efficient conversion between normal on-chip devices and the SPP T-line, one needs to match both impedance and momentum within a broadband for (sub)-THz signal propagation. The polarization of guided wave must be transformed to TM-polarized supported by SPPs as well. Such a design is illustrated in Fig. 4a, which is characterized by a gradient grooves and a GCPW with a smoothly flaring ground in a linear format. Such a symmetrical SPP structure with the same corrugation grooves on both sides of the strip could also support the SPP modes efficiently and has been demonstrated in ref. 9 for GHz applications. As discussed in ref. 15, this symmetrical SPP modes oscillates and interact with each other, while create two mode (namely, odd mode and even mode) for propagation. Compared with the anti-symmetric mode (odd mode, corresponding to out-of-phase oscillation), the symmetric mode (even mode, corresponding to in-phase oscillation) has been found to be less sensitive to loss. The dispersion curves bending for odd modes start at much higher frequencies that are basically not considered in on-chip communication. As such, only the even mode is discussed throughout the remaining of paper. The ground plane has been well defined, which is implemented underlying the SPP T-line for preventing the field from penetrating into the high index lossy substrate, as discussed above. Accompanying with CPW, now the full ground plane forms a GCPW configuration at the transition part. The dispersion property of SPPs maintains for such implementation but the slow-wave effect is slightly weaken. As such, a deeper groove is preferable to obtain stronger field confinement but cannot be arbitrary large due to the propagation length concern. The groove depth *h* is kept in the range of one or two tens of μm to preserve at least 10-wavelength long propagation length, which is normally required for constructing silicon-based channel. Note that the gradient corrugation structure reported in ref.^13^ cannot generate wideband 50 Ω impedance on-chip, but a much smaller width is desirable which is normally 5 μm for a 3.3 μm thick copper metal when the junction part is realized by microstrip line. The connector interface design in ref. 14 involves multiple turning points, potentially introducing additional loss for the ultra-thin metal in CMOS. To overcome this problem, a gradual groove evolving from *h* = 0 μm to *h* = 12 μm is proposed, thus avoiding any abrupt conversions at the interface. The grooves depth of 0 μm renders the SPP T-line nothing more than a bare on-chip microstrip line, and the line width of 5 μm is well matched to the optimum for 50 Ω impedance over very wideband. With co-planar ground implemented by the same level copper metals with a 7 μm separation to the microstrip at each side, the transition part has a simulated impedance matching to 50 Ω up to 2.5 THz.

While the groove depth is linearly increased to smoothly transform the momentum of microstrip line (*k*_*0*_) to the SPP T-line (*k*_*x*_), the flaring ground in a linear format overcomes the mismatch between the GCPW and SPP T-line structure. At the injection part, the two sides ground of CPW help confine the *E*-field between the microstrip line and them, achieving high transmission efficiency of TEM wave. As the grooves becomes deeper, the TEM modes gradually transform to the bounded mode, but the associated slow-wave effect is not significant, rendering the ground of CPW remain necessary. Note that, from the dispersion property, for a given frequency the confinement of *E*-filed inside grooves mainly depends on the grooves depth (when the groove periodic is fixed). For a shallow grooves the bounded modes are loosely confined and the TEM mode are not completely converted to the bounded mode. As such, the ground of CPW needs to be linearly regulated away from the grooves so that the incomplete conversion of TEM mode remains effectively confined by the CPW. As the groove depth becomes sufficiently deep for efficient SPP modes transmission, the two sides ground of CPW are faded out sharply. To avoid the exponential regulation reported in ref. 13, 14, 15, instead a linear format is proposed here to mimic its effectiveness at THz in CMOS. The periodic pitch *d* is chosen as 15 μm while the gap of groove *a* is fixed at 2.4 μm, which is the minimum metal separation constrained by the design rule. The loss tangent of SiO_2_ (*ε*_*r*_ = 4) covering the structure is 0.001. Denote the grooves evolution rate as *k*, and *C*_*x*_, *C*_*y*_ is the distance between the initial point of the ground of CPW to the one that the CPW becomes abruptly faded out, as illustrated in Fig. 4a. A smaller *k* means a more abrupt changing of grooves depth, while a smaller *C*_*y*_ indicates slow fading of ground. For example, *C*_*y*_ = 0 μm corresponds to a pure GCPW without any regulation, while *C*_*y*_ = ∞ indicates a immediate faded-out ground of CPW. As such, an optimum may exit for either *k* or *C*_*y*_. The gap between the bared microstrip line to the ground of GCPW is kept as 7 μm to obtain sufficiently high transmission efficiency and impedance matching for TEM mode at the injection interface. The effectiveness of mode conversion could be evaluated by the *E*_*x*_ component as shown in Fig. 4b captured on the *xy* plane. Here, the gradient factor *k* is chosen as 1(μm), and *C*_*x*_  = 185 μm while *C*_*y*_ = 10 μm, respectively. As observed, the *E*-field are highly localized in a very tiny region and propagate with low loss for the double side deep corrugated region, while it becomes diluted inside the CPW area, i.e., a apparently large area of *E*-field observed for the CPW waveguide. This attributes to the fact that the eigenmodes of CPW and the plasmonic waveguide are quasi-TEM and quasitransverse electromagnetic (TM) respectively, and the propagation wavelength of the plasmonic waveguide is much higher than that of CPW or microstrip line. In the GCPW region, traditional guided waves are supported, leading to the *E*-field mainly polarized to the *y*-direction and confined by the ground of CPW. The resulting *E*-field vector on the CPW has small *E*_*x*_, while it becomes significant on the plasmonic waveguide. However, a smooth conversion of *E*_*x*_ between the two regions could be observed as well. The *E*_*x*_ components gradually increases from the GCPW region to the plasmonic region along with deeper the groove, and reaches a maxima next to the region where GCPW abruptly vanishes. This also corresponds to the place that the groove depth *h* reaches its maxima. The same scenario could be found for the quasi-TM mode to quasi-TEM mode conversion as shown in Fig. 4c. Such a gradient conversion demonstrates the effectiveness of mode conversion by incorporating the proposed linearly fading ground with the gradient groove.

**Figure 5:**
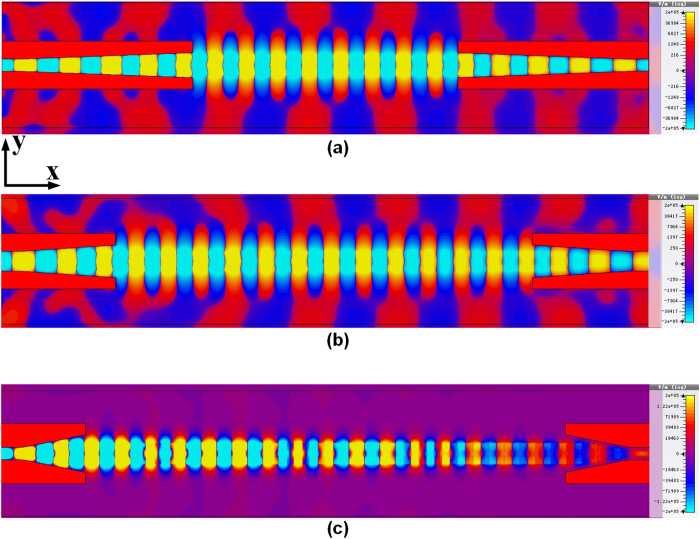
The simulated near-field result of *E*_*y*_ component evaluated at the *xy* plane for the proposed surface plasmonic waveguide with GCPW converter structure: (**a**) *k* = 0.5 μm and *C*_*y*_ = 10 μm, (**b**) *k* = 1 μm and *C*_*y*_ = 10 μm and (**c**) *k* = 2 μm and *C*_*y*_ = 20 μm. Note that for large *k*, *C*_*y*_ needs to be increased accordingly so that the GCPW always keeps sufficient distance away from the plasmonic waveguide.

It is also interesting to investigate how the GCPW impact the mode conversion. Figure 5 illustrates the *E*-filed distribution (*E*_*z*_ component) evaluated at the *xy* plane for the whole SPP T-line including conversion parts at 2 THz, with the same color scale. The right side of converter is simply the replica of the left one, and the conversion is done for both TEM-to-SPPs and SPPs-to-TEM. In contrast to the designs in the microwave region, the *E*_*z*_ component of the conventional microstrip line or CPW now exhibits significant in mm-wave to THz^39^,^40^,^41^. In the presence of dielectric the TEM mode is supported only at the zero frequency, while in THz region no TE modes or TM modes are pure. This leads to a hybrid eigenmodes solved for microstrip line and CPW structure, which cannot be neglected in THz frequency. As such, the hybrid-modes solutions have a *z* dependence of *e*^−*jkz*^ 39. Besides, due to the non-perfect conducing nature of the ground plane (especially for the signal injection regions), the surface-wave can exit and propagate on the surface of the ground plane^42^,^43^,^44^, and parts of the radiated energy are reflected. All these effects makes the conversion difficult, however, as the guided waves are mainly confined by the well-defined ground (especially for GCPW structure) in this design, we could still assume a quasi-TEM excitation for conventional THz waveguide^45^,^46^. As a result, the mode conversion simplifies to the quasi-TEM mode to TM mode conversion, at the mean time the hybrid modes with various wave vectors are transformed to TM mode via gradual momentum adaptation. Simulated at 2 THz and shown in Fig. 5a–c, when a plane TEM wave is injected into the left edge of the structure, the *E*_*z*_ components for GCPW part are significant, verifying the above discussion. Meanwhile, the CPW for all cases efficiently direct the quasi-TEM wave with *E*-field confined between the bared microstrip lines to the ground of CPW, while the field confinement profiles of the bounded mode significantly varies with *k*, i.e., by changing the gradient factor *k*, the conversion efficiency varies obviously. Here, the vertical separation *C*_*y*_ is kept constant of 10 μm while the horizontal distance *C*_*x*_ needs to be scaled accordingly such that the ground of CPW always sharply vanishes as long as *h* reaches its maxima. Illustrated in Fig. 5a in which the gradient factor *k* = 0.5, during conversion the *E*-field of guided modes are fundamentally restricted by GCPW with low propagation loss while they converts to the TM polarized mode and highly confined by the deep scaled subwavelength grooves with very small amount of field radiated outward the grooves. The EM wave thus propagates in the form of polarized SPP modes with low loss, which has a near-field huge *E*_*z*_ component. After traveling through several wavelength the SPP modes are gradually transformed back to quasi-TEM mode for receiving with the same process. The physical length of the whole structure is 1 mm. As such, the proposed SPPs waveguide with mode converters demonstrates a transmission efficiency higher than 90% that is hardly achieved by conventional waveguide structures such as GCPW (shown later) at THz frequency. The loss sources could be attributed to metal intrinsic Ohmic loss, dielectric absorption loss (due to interband damping of interactive SPPs^47^), and conversion loss arisen from incomplete conversion from hybrid modes to SPP modes or vice versa. Fortunately, all these three losses could be well governed as they are geometrical dependent on the structure of SPP waveguide. For instances, by widening the line width *w*, both Ohmic loss and propagation loss by absorption can be greatly suppressed, while a deeper grooves fundamentally trade-off propagation length. Meanwhile, the conversion loss due to the gradual change of groove depth decreases with a smoother gradient factor *k*. To verify this, Fig. 5b,c illustrates another cases of conversion with *k* = 1 and *k* = 2, respectively. Similarly, the guided wave presents a confinement by the GCPW during conversion and subsequently transformed to the bounded modes which exhibits remarkably subwavelength effect and field confinement in the plasmonic waveguide part as well.

For a metallic waveguide surrounded by non-absorbed dielectric (with dielectric constant of *ε*_2_) and conducting medium (with dielectric constant of *ε*_1_), the wave vector of SPP mode propagating at the interface along the *x* direction (*e*^*jβx*^) between the two half spaces can be described by 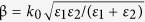, and the expression is valid for both real and complex *ε*_1_. In low frequencies (mm-wave or lower), SPP modes propagation constant is close to *k*_*0*_ at the light line, and the surface waves extend over multiple wavelengths into the dielectric space. In this regime, SPPs therefore are more akin to a grazing incidence light field that exhibits a highly delocalized nature. As the frequency goes up, the wave vector increases while the group velocity decrease, accompanying the excitation of SPP modes. For real metal with loss, the dispersion of surface plasmon is now dependent on the metal skin depth *l*_*s*_ due to the finite conductivity of metal. In this case, the wave vector cannot be infinitely large (the case of metal being treated as PEC) and the SPPs propagation length is related to the skin depth as well. To achieve confinement at THz with small plasmon wavelength, the resonance figure of merit *l*_*s*_/*a* should be small, which demands a larger *a*^48^. To further decrease diffraction, the groove period *d* should be small as well. This nonetheless tradeoffs the propagation length that is proportional to *d*^*2*^ 49.

**Figure 6:**
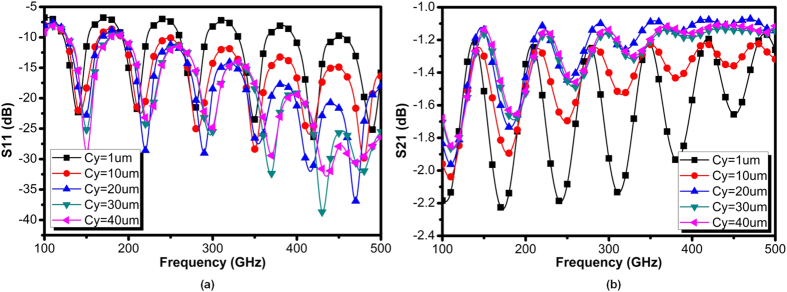
(**a**) Simulated input reflection coefficient (S_11_) of the designed on-chip SPP T-line with converter for different *C*_*y*_ with *d* = 12.4 μm, *a* = 2.4 μm, *w* = 5 μm, and (**b**) simulated transmission coefficient (S_21_) of the designed on-chip SPP T-line with converter for different *C*_*y*_.

**Figure 7:**
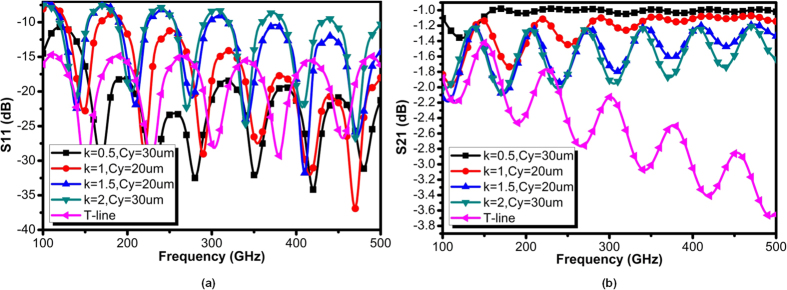
(**a**) Simulated input reflection coefficient (S_11_) of the designed on-chip SPP T-line with converter for different *k* with optimized *C*_*y*_ for each cases, and (**b**) simulated transmission coefficient (S_21_) of the designed on-chip SPP T-line with converter for different *k* with comparison to conventional on-chip transmission line (T-line).

Owing to the thin film metal strip with centralized symmetrical grooves, the SPP modes supported by dual side of grooves and will interact with each other. Besides, the surface plasma acquires electrostatic characteristic only if the frequency reaches *ω*_*sp*_. Below this threshold frequency the SPP modes are not bounded entirely insides the grooves but instead mutually overlaps with each other. As such, the transmission of SPPs will present a “damping” as long as the operation frequency is not sufficiently closed to *ω*_*sp*_. Note that this phenomenon commonly occurs in plasmonic waveguides, regardless of the existence of the mode converter. However, without converters the transmission of plasmonic waveguides degrade due to impedance and momentum mismatches. To evaluate how the converter affects the transmission, Figs 6 and 7 illustrate groups of simulated reflection and transmission for the structure shown in Fig. 4a, with *C*_*y*_ and *k* as variables. As observed, the converter behaves more effective at high frequencies region, in which the reflection coefficients (S_11_) are below −10 dB, demonstrating low reflection loss. Moreover, as shown in Fig. 6a, the conversion by *C*_*y*_ = 1 μm is not effective as it does not guarantee good impedance matching for conventional quasi-TEM modes, as expected. By increasing *C*_*y*_, the ground of CPW linearly vanishes as the rate of gradient groove, and the CPW always matches the impedance of the quasi-TEM mode in the hybrid-mode region. An over large *C*_*y*_ is not desired, as it fades out faster than the grooves, while an intermediate could be an optimum for impedance matching. With *k* = 1, the optimal *C*_*y*_ is found to be 30 μm. The transmission coefficient (S_21_) is illustrated in Fig. 6b, in which multiple ringing region could be observed. In low frequency region, the ringing attributes to the combination effects of reflection and modes interaction as described before. By increasing *C*_*y*_, this ringing obviously alleviates at low frequency region as the reflection reduces. However, further increasing *C*_*y*_ provides a marginal improvement on transmission. All this observation, on the other hand, verifies that the proposed CPW improves the matching of impedance during the transition. In fact, the shape or size of the CPW should not affect the SPP dispersion. As a result, the momentum matching and polarization are exclusively governed by the gradient groove. Figure 7(a,b) illustrates the reflection and transmission under various gradient factor *k*. As *C*_*y*_ between 10 μm and 40 μm presents most effective impedance matching, only 20 μm or 30 μm are considered in these two figures. As illustrated, *k* ≥ 1.5 corresponds to degraded reflection and transmission, while *k* = 2 exhibits strongest reflection and ringing. The case of *k* = 1 is somewhat inferior to that of *k* = 0.5, in terms of magnitude and ringing, but greatly better than that of *k* = 1.5. It can be therefore confirmed that the ringing in low frequency region for *k* ≥ 1.5 is simply due to reflection, while in the high frequency part attributed to the more rapid changes of *k*. It seems that an abrupt increment of *k* can only partially achieve mode conversion, and the hybrid modes may even prevail in the plasmonic part. The incomplete mode conversion directly leads to not only reflection due to momentum mismatch but also mixed polarization exit for transmission. As such, they preserves the polarization of conventional quasi-TEM with lower transmission efficiency. Note that the fringing pattern in Figs 6 and 7 could also be attributed to the impedance mismatch between the plasmonic waveguide and surrounding medium. The SPPs with propagation constants β between the light lines of air and the higher-index substrate (Fig. 2c) are excited inherently in form of leaky wave. The loss of SPP energy is in part due to the inherent absorption inside the metal, but also because of leakage of radiation into surrounding medium. The surface wave coupling therefore becomes less effective when the reflected wave destructively interferes with radiation wave that commonly observed in optics, such as grating coupling. However, all cases of transmission for the proposed plasmonic waveguides are still much better than the transmission line at high frequencies, which clearly degrades with frequency. From comparison, the transmission line already matches to 50 Ω impedance in a broadband, so its reflection is negligible. Even with the underlying ground shielding, the conventional T-line still suffers from high loss in sub-THz realm, implying the high potential of plasmonic waveguide as silicon channel. In conclusion, the conversion part favors smooth conversion corresponding to low *k* and proper *C*_*y*_.

### Mode converter without flaring ground

With the proper excitation, surface plasmons can be established to build functional devices^50^,^51^,^52^,^53^,^54^,^55^,^56^,^57^,^58^,^59^,^60^,^61^. The above plasmonic waveguide with a mode converter demonstrates high conversion efficiency in silicon. However, due to the huge size of CPW it is extremely area consuming and therefore not desired in CMOS integration. For example, the design shown in Fig. 4a with *k* = 0.5, *C*_*y*_ = 30 μm, *C*_*x*_ = 185 μm corresponds to 0.015 mm^2^ silicon area only for one channel. In typical I/O with aggregated bandwidth toward Tera-scale, nearly thousands of cores needs to be integrated on chip and the number of the resulting interconnect would be geometrically increased as well^62^. This situation unfortunately cannot be tolerant in the existence of CPW. Moreover, in sub-micron CMOS especially in 65 nm and below technology nodes, only diagonal routings are allowed due to the stringent design rule. In other words, *C*_*y*_ is constant which is far away from the optimum. This in turn renders the plasmonic waveguide design with GCPW grounding not attractive.

**Figure 8:**
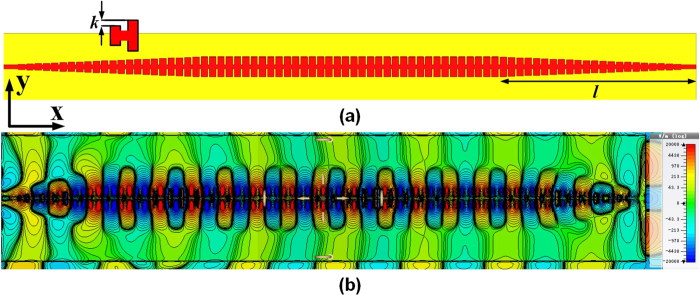
(**a**) The proposed SPP T-line with mode converter structure featured by gradient grooves, while parameter *k* denotes the gradient factor, (**b**) the simulated *E*_*x*_ component evaluated at the *xy* plane demonstrating the mode evolution by the proposed converter structure.

**Figure 9:**
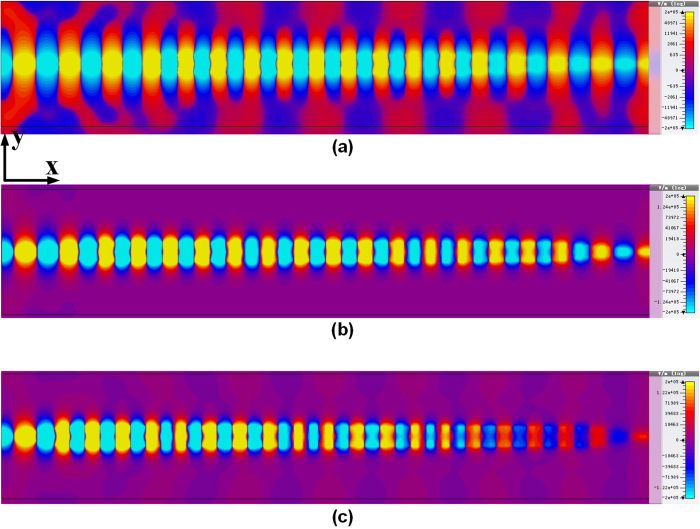
The simulated near-field result of *E*_*y*_ component evaluated at the *xy* plane for the proposed SPP T-line with gradient groove converter structure. (**a**) *k* = 0.5 μm, (**b**) *k* = 1 μm, and (**c**) *k* = 2 μm.

Recall that the GCPW structure mainly contributes to the impedance matching but rather the conversion of the polarization, the plasmonic structure could be further simplified by omitting the GCPW part, while the line width *w* needs to be tuned accordingly to maintain impedance matching at low frequencies. With the line width of 5 μm, the reflection coefficient could reach −10 dB and below^1^. More importantly, the mode conversion can be still realized by the gradient groove, which is easy to implement on-chip. Such a design is depicted in Fig. 8a with a copper metal M1 as the ground plane as well. Here, the conversion efficiency depends only on gradient factor *k* and line width *w*. Figure 8b illustrates the *E*-field (*E*_*x*_ component) distribution for the simplified version evaluated in the *xy* plane at 2 THz. Similar to the observation in Fig. 4b, the *E*_*x*_ component starts with weak amplitude but gradually increases to its maxima for transmission over a long distance. At the TEM wave injection interface the structure degenerates to a microstrip line whose impedance is mainly governed by the line width while it supports quasi-TEM waves with a wave vector *k*_*0*_. As the guide modes are bounded to either the metal surface or surrounding ground, the low frequency transmission is expected to be degraded. By gradually enlarging the groove depth *h*, the quasi-TEM modes are smoothly transformed to the bounded mode with larger *k*_*x*_, and the resulting *E*_*x*_ component is significant demonstrating the tight confinement of SPPs. The SPP modes are now highly localized by the grooves with short decaying length, and propagate with low loss. The receiving part simply decrease the groove depth in a linear manner, so that the bounded modes are converted back to quasi-TEM modes for receiving by subsequent 50 Ω system. The above observations are further verified by Fig. 9(a–c) illustrating the simulated near field results for the proposed compact mode converter. They have similar conclusions as that of Fig. 5(a–c), in which the conversion efficiency could be obtained by a smaller *k* as well.

**Figure 10:**
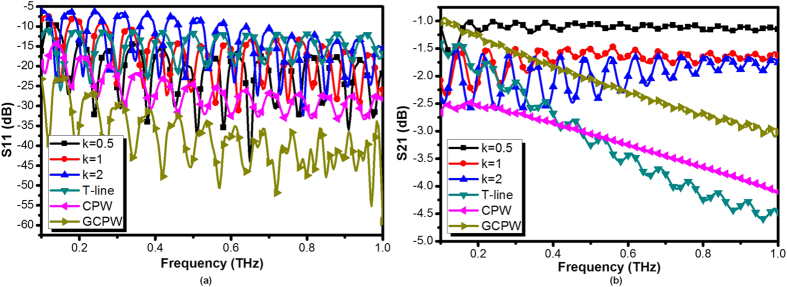
(**a**) Simulated input reflection coefficient (S_11_) of the designed on-chip SPP T-line with converter for different *k* with *d* = 12.4 μm, *a* = 2.4 μm, *w* = 5 μm, and (**b**) simulated transmission coefficient (S_21_) of the designed on-chip SPP T-line with converter for different *k*. Conventional waveguide T-line (microstrip), CPW and GCPW are incorporated as well for comparison.

The reflection and transmission of the proposed SPP T-line with the converter are illustrated in Fig. 10 with comparison to the three dominant on-chip interconnect structures, namely, T-line, CPW and GCPW, all have been designed and optimized for mm-wave to THz applications. All conventional structures supporting quasi-TEM modes clearly demonstrates wideband impedance matching up to 500 GHz. For plasmonic waveguides only those with a small gradient factor *k* have similar performance with conventional waveguides, in accordance with the results found in above study. As such, by tuning the line width *w* the on-chip plasmonic waveguides can avoid the use of CPW as conversion. With broadband impedance matching, the transmissions could be fairly compared. First, compared with Fig. 6b, the plasmonic waveguide suffer from a slightly increase of loss in the low frequency region mainly due to impedance mismatch, as expected. For example, the case with *k* = 1 for the design with CPW has an insertion loss of −2 dB at 100 GHz, while it decreases to −2.5 dB for the new design, as expected. For *k* = 0.5, however, only 0.1 dB drops at around 120 GHz, demonstrating high conversion efficiency for both designs. For *k* > 1, the two designs experiences similar results as they both encounter impedance mismatches at same extent in low frequency. Interesting to note that, the transmission of plasmonic waveguide are not necessary better than conventional counterparts, while they are obviously much better as frequency goes up, as long as impedance/momentum matching attained. With slower gradient increment in the conversion part, the transmission efficiency is found to be better over a very broad band. These observation demonstrates the wideband low loss transmission by SPP T-line even with TEM wave injection and receiving. The proposed SPP T-line with converters demonstrates high and flat transmission coefficient from 400 GHz to 1 THz, and no transmission degradation found in high frequency region. Among traditional waveguide the GCPW structure has relatively low loss compared to CPW and T-line as it inherently acquires better field restriction among grounds. However, it is sensitive to skin and proximity effects in high frequency for which the current density is large with severer Ohmic loss. The current tends to crowd on the metal surface with more energy radiated outward into the dielectric. These in turn show the leaky field confinement of conventional waveguide structures and therefore they are not desired in (sub)-THz.

**Figure 11:**
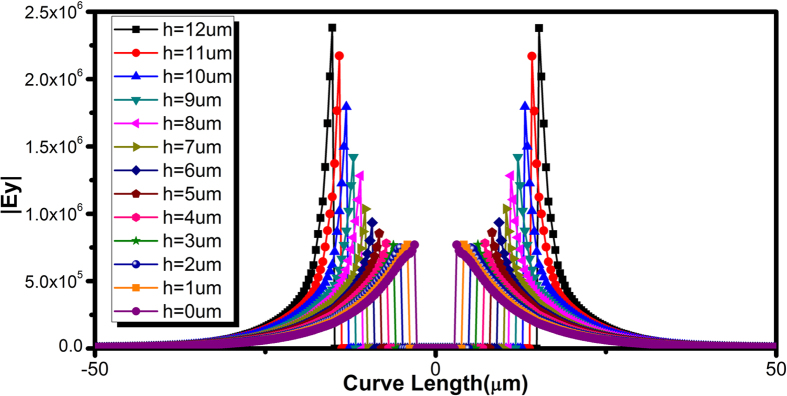
Simulated *E*-field enhancement along the vertical cut for the converter part demonstrating mode conversion achieved by the gradient groove technique.

**Figure 12:**
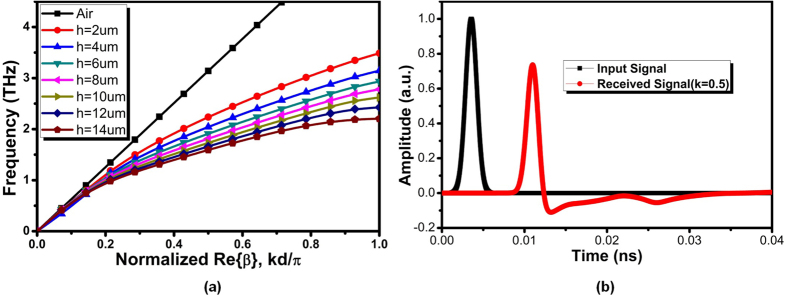
(**a**) The simulated dispersion diagram with different groove depth *h* ranged from 2 μm to 14 μm in support of the mode conversion by gradient groove demonstrated in Fig. 11, and (**b**) Time-domain SPP signal supported by the dual side strips structure under the input of a Gaussian pulse with broadband signals from 0 to 500 GHz.

The success of mode conversion can be observed by Fig. 11, in which the details of confinement can be clearly observed from *E*-field enhancement in the cross-sections perpendicular to the strips. Here, we draw a straight line across the grooves and evaluates the |*E*_*y*_| components. Clearly, the *E*-field strength linearly increases with grooves depth *h*, demonstrating a gradual mode conversion from weak SPPs to the bounded modes. All traces are symmetrical with respect to the geometrical center of SPP T-line as dual groove structure are developed in this design. With *h* = 12 μm, the field enhancement reaches its maxima illustrating tight field confinement by the grooves, while it drops linearly as groove depth decreases. Interesting to note that when *h* drops to 4 μm or below the |*E*_*y*_| components stop decreasing any more. This scenario could be expected as the quasi-TEM prevails among the hybrid modes during initial transition, and the *E*-filed are loosely localized with very weak field enhancement. To evaluate how the groove depth affect the dispersion, Fig. 12a illustrates the simulated dispersion diagram. With deeper the grooves, the dispersion of SPP T-line is more bending away from the light line (*k*_*0*_ = 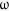/c) with lower asymptote frequency, demonstrating highly field confinement for deep groove while loose confinement for shallow grooves. Note that with *h* = 0 the SPP T-line eventually degenerates to a bare microstrip. Therefore, the loose field confinements are naturally more akin to quasi-TEM, rendering the mode conversion starts from a shallow groove avoiding huge momentum mismatch at the injection interface. As the proposed SPP T-line is wideband with low loss, a Gaussian pulse with spread spectrum covering from DC to 500 GHz is inputted to one terminal of the proposed SPP T-line, as shown in Fig. 12b. As observed, the transmitted SPP signal maintains almost the same waveform as the input demonstrating low distortion that may mainly attributes to reflection and dispersion. Owing to the natural cut-off frequency at asymptote frequency, as shown in Fig. 12a, the SPP T-line demonstrates a very sharp decline of transmission forming a bandgap region in which the SPPs acquire electrostatic character. This property helps shape the channel characteristic to a well-defined model for on-chip integrated wireline transceiver design where the equalization parts could be significantly simplified resulting in both power and area reduction.

### Measurement Results

**Figure 13:**
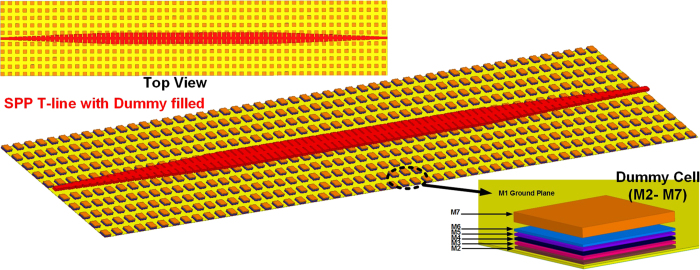
The simulation approach by considering the dummy metal fill effect with illustration of dummy configuration.

**Figure 14:**
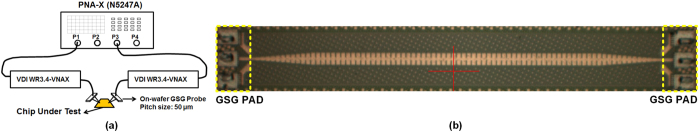
(**a**) Measurement setup for the (sub)-THz SPP T-line with converter, (**b**) die micrograph of the proposed SPP T-line with converter in 65 nm CMOS for Terahertzes applications.

The proposed SPP T-line with converter is designed and fabricated in standard 65 nm CMOS technology, in which eight copper metals and one Alumina metal are available for routing. To mimic the real scenario after manufacturing by CMOS technology, the prototype is simulated first as shown in Fig. 13, and the length of line is 1.05 mm. The groove gradient factor *k* is chosen as 1(μm) for this design, while it could be further reduced to 0.5 or below in the future design in which the length of interconnect is much longer. The top copper metal with thickness of 3.3 μm is exclusively employed for constructing the whole structure. With bottom metal M1 as ground plane, the metal dummy containing M2–M7 in stack are required to surround among the structure in order to fulfill the metal density rule. A short description of the dummy effect is introduced later. The capability of confining EM fields and efficient conversion can be evaluated by measuring the reflection coefficient S_11_ and the transmission coefficient S_21_. The (sub)-THz TEM signal is injected from one terminal into the proposed SPP T-line, and the resulting *S* parameters will present both the conversion/transmission efficiency. The proposed structure is measured on CASCADE Microtech Elite-300 probe station and Agilent PNA-X (N5247A) with the VDI providing signal source from 220–325 GHz. Connectors, probe, waveguides, cable loss as well as Ground-signal-Ground (GSG) Pad are well calibrated for all available bands before on-wafer probe testing. Such a calibration scheme will be elaborated in the next section. Due to limited equipment available, the measured frequency can be up to 325 GHz at this moment. The measurement setup is shown in Fig. 14a, and the prototype die photo is shown in Fig. 14b. The design parameters are as followed: periodic pitch *d* = 12.4 μm, groove depth *h* = 12 μm, groove width *a* = 2.4 μm, and line width *w* = 6 μm. To prevent significant electromagnetic coupling between the two PADs and the core device, the microstrip parts at both sizes are slightly longer than those in previous simulation, and a finite phase shift could be expected.

**Figure 15:**
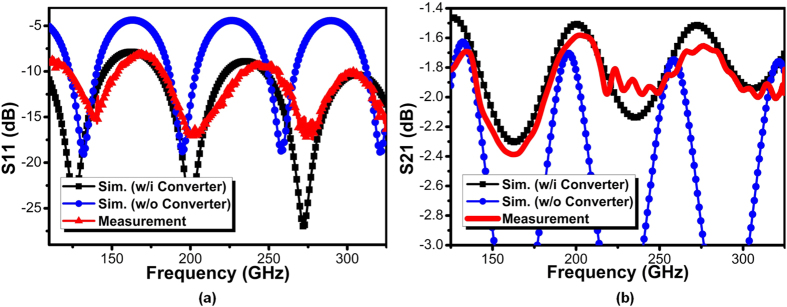
Measured and simulated *S* parameter results: (**a**) the measured and simulated results of the input reflection coefficient (S11) for the proposed SPP T-line w/i and w/o converter, and (**b**) measured and simulated results of the transmission coefficient (S21) for the proposed SPP T-line w/i and w/o converter.

The well matching of both momentum and impedance between conventional microstrip and the plasmonic waveguide serves to a key foundation of effective measurement with capability of accurate calibration. To verify the proposed structure experimentally, *S* parameters measurement results are shown in Fig. 15 with comparison to simulation results (including design with and without converter). Good agreements between simulation and measurement results could be observed from 110 to 325 GHz. We notice that the input reflection coefficient (S_11_) is nearly lower than −10 dB for all bands while only in the range of 160–175 GHz it degrades to −9 to −8 dB, which is still tolerable for on-chip interconnection. However, the result in Fig. 15a still demonstrates the effectiveness of mode conversion by the proposed gradient groove technique even without the fading CPW introduced in Fig. 4a. In contrast, the plasmonic waveguide without converter presents huge reflection. It suffers from 5 dB reflection loss, indicating 32% electromagnetic waves are reflected at the signal injection junction. The transmission coefficient (S_21_) compared with simulation results are given in Fig. 15b which shows good agreement as well across wideband. The transmission of SPP T-line maintains high efficiency from low to high frequency with only 1.9 dB averagely loss, demonstrating the fundamental guiding property of SPPs described above. Both simulated and measured S_21_ do not degrade when frequency rises up, which is in contrast to conventional waveguide in CMOS (illustrated in Fig. 10b). This in turns demonstrates the highly potential of the proposed surface plasmonic waveguide to replace conventional waveguide as future on-chip interconnects. Due to degraded reflection from 140–180 GHz, the transmission is therefore not optimized within the same area. With 10% signal reflection, the corresponding transmission drops 0.4 dB lower than average. Fortunately, as stated in the previous section, by adjusting the line width *w* and reducing groove gradient *k*, the reflection could be effectively attenuated as well, leading to higher transmission efficiency over wideband with flat response. However, the plasmonic waveguide without converter shows noticeable transmission loss (>3 dB) mainly due to large reflection. Though a little bit transmission degradation compared to the structure in Fig. 4, the novel converter design in Fig. 8 does not need GCPW but can still maintain the high conversion efficiency for the whole surface-wave I/O design in a much more compact area. In conclusion, a mode converter with high conversion efficiency is necessary to build on-chip plasmonic interconnects.

## Materials and Methods

### Simulation of SPP T-line with converter

In much contrast to board level design, the passive elements are found to be difficult in simulation for on-chip realization in CMOS especially at frequency toward mm-wave and beyond. One design example is depicted in Fig. 13 in which a rich of metal dummy are filled surrounding the core device that could be further observed in the die photo of Fig. 14b. Such a dummy fill process (density > 20%) is a must before fabrication in order to minimize metal thickness variation especially for long routing. However, they may introduce additional electromagnetic coupling and loss at high frequency. To mimic such effect, the dummy cell containing copper layer M2 to M7 are in stack and periodically arranged as shown in Fig. 13. In reality the dummy cell could be as small as 1 μm^2^ which will dramatically increase the simulation burden. Instead, we use the method introduced in ref. 62 while all dummy metals in stacked are not connected to each other through vias. This ensure all dummy cells are opened and their mutual coupling are electromagnetically minimized. As such, the size of 10 μm × 10 μm are ultimately chosen for dummy cell to minimize the simulation burden while maintain their effective impact on the results. The proposed structure is simulated by full-wave near-field simulation using finite-difference-time-domain (FDTD) method embedded in commercial EM simulator CST Studio. The boundary condition is set as open to simulate the real space. The boundaries are at large distances from the metal structure as well to avoid significant reflection.

### On-chip implementation of SPP T-line with converter

To verify above design observations, a SPP T-line with proposed mode converter was fabricated by 1P9M bulk 65 nm CMOS process with the die micrograph shown in Fig. 14d. Including GSG Pad at both sides, the area occupation is 150 μm × 1350 μm, while the core device consumes approximate 150 μm × 1050 μm area. The spacing between two adjacent groove a is chosen as 2.4 μm which is the minimum metal gap allowed by the design rule. Increasing a will notably degenerate the bound modes that leads to higher transmission loss. The top Aluminum layer (Metal 9) with 1.325 μm thickness is used to form the Ground-Signal-Ground (GSG) PADs which has measured characteristic impedance around 50 Ω across 110–325 GHz frequency range. Note that the thickness of metal has very limited influence on the dispersion relation of SPPs because the sub-wavelength nature still maintains (the *H*-field remains unquantized in the *x* direction.)^9^, while the resistive loss could be much reduced for a thicker metal. For SPP T-line the intrinsically resistive loss cannot be omitted by realizing with a better confinement. Two terminals of the SPP T-line with converter are directly connected to the signal trace of PAD which has the same width of 6 μm as that of microstrip part of converter to avoid further conversion and loss.

### Calibration of Measurement Results

The calibration of core device removes the effects of unwanted portions of the structure that are embedded in the measured data by subtracting their contribution. Connectors, probe, waveguides and cable loss are well calibrated first before on-wafer probe testing. However, the influence of GSG PADs cannot be neglected any more. The equivalent model of PAD by lumped elements could be found in ref. 63, and the extracted capacitance seen by the core device could be as high as 45 f F in 300 GHz which have already introduced noticeably loss to the core device. As such, the contribution of PAD must be well calibrated as well. Before measuring the *S* parameter of the proposed plasmonic waveguide, we simulate and measured the *S* parameter of PAD by arranging them face-to-face connected to each (not shown here). As a result, when measuring the prototype shown in Fig. 14b the actual *S* parameter measurement result is the combination of core device plus two PADs. The total T-parameter^64^,^65^ could be described by matrix [T_toal_] = [T_p_][T_DUT_][T_p_], where [T_p_], [T_DUT_] denotes the T-parameter of PAD and core device (device under test), respectively. As such, the T-parameter of the core device could be expressed by


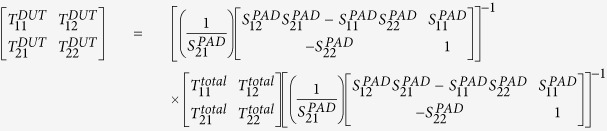


And the final *S* parameter of the core device can be obtained:


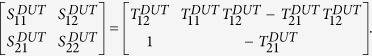


In general, this calibration process is quite fit for low frequency measurement. In high frequency range such as 220–325 GHz, the signal Gen typically generates non-constant output power during measurement, which makes the high frequency measurement quite challenge. This situation becomes worst at the edge of frequency sweep of equipment. Other effects such as non-perfect probing or instantaneously perturbation would lead to noticeable variation of measured results. Therefore, a large amount of samples are required and averaging or smoothing are normally needed during data processing.

## Additional Information

**How to cite this article**: Liang, Y. *et al*. On-chip sub-terahertz surface plasmon polariton transmission lines with mode converter in CMOS. *Sci. Rep.* **6**, 30063; doi: 10.1038/srep30063 (2016).

## Figures and Tables

**Figure 1:**
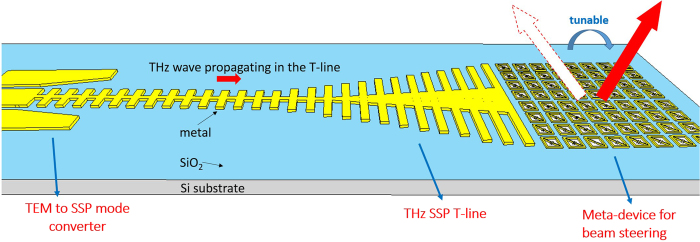
A CMOS THz communication I/O link, which is fed by THz spoof-surface-plasmon (SSP) THz transmission lines (T-line). While on-chip oscillator network can only generates TEM-wave source, the high output power of beam steering antenna relies on a highly efficient mode conversion to transform the TEM mode to SS mode with low loss in a wide band. The proposed converter is featured by a linearly flaring GCPW with gradient groove.
